# Involvement of the Autophagy-ER Stress Axis in High Fat/Carbohydrate Diet-Induced Nonalcoholic Fatty Liver Disease

**DOI:** 10.3390/nu12092626

**Published:** 2020-08-28

**Authors:** Xiu Zhou, Sherouk Fouda, Dongli Li, Kun Zhang, Ji-Ming Ye

**Affiliations:** 1 School of Biotechnology and Health Sciences, Wuyi University, Jiangmen 529020, China; zhou.xiu@hotmail.com (X.Z.); wyuchemldl@126.com (D.L.); kzhang@wyu.edu.cn (K.Z.); 2 International Healthcare Innovation Institute, Jiangmen 529040, China; 3 School of Chemical Engineering and Light Industry, Guangdong University of Technology, Guangzhou 510006, China; 4 School of Health and Biomedical Sciences, RMIT University, Melbourne, VIC 3083, Australia; sherouk.fouda@rmit.edu.au

**Keywords:** nonalcoholic fatty liver disease (NAFLD), nonalcoholic steatohepatitis (NASH), endoplasmic reticulum (ER) stress, autophagy, inflammation

## Abstract

Nonalcoholic fatty liver disease (NAFLD) is the most common chronic liver disease that can progress from simple hepatic steatosis to nonalcoholic steatohepatitis (NASH), and even further to liver cirrhosis or liver cancer. Overconsumption of high fat and/or carbohydrate are among the most common lifestyle factors that drive the development and progression of NAFLD. This review evaluates recent reports on the involvement of autophagy and endoplasmic reticulum (ER) stress in the pathogenesis of NAFLD. Here, we reveal a mechanism of an intrinsically linked axis of impaired autophagy and unresolved ER stress that mediates the development and progression of NAFLD resulting from the overconsumption of high fat and/or carbohydrate.

## 1. Introduction

Nonalcoholic fatty liver disease (NAFLD) is the most common chronic liver disease, characterized by excessive lipid accumulation in the liver in the absence of significant alcohol consumption or liver infectious diseases [1]. This metabolic disease starts from simple fatty liver (hepatic steatosis), which may advance to nonalcoholic steatohepatitis (NASH) and even further to liver cirrhosis or liver cancer [2,3]. Currently, the prevalence of NAFLD is more than 25% worldwide and it has imposed a serious health burden on the society [4]. Although hepatic steatosis per se is relatively benign and reversible, 8–20% of hepatic steatosis progresses to NASH with additional hepatic inflammation, injury and even fibrosis [1,5]. Approximately 15–25% of NASH progresses to irreversible liver cirrhosis within 10 years and some can advance to liver cancer, possibly due to deregulated cell regeneration in the process of the repairing cell damage [1,4].

Recent laboratory studies suggest that a high intake of fat and carbohydrate can induce dynamic changes in autophagy and endoplasmic reticulum (ER) stress in liver [6,7,8,9,10,11]. Therefore, this review evaluates the literature reports on the possible roles of autophagy and ER stress in the development and progression of NAFLD due to dietary high fat or high carbohydrate (fructose or sucrose).

## 2. How High Fat/High Carbohydrate Consumption Induces NAFLD

Although genetic predisposition has been suggested to influence the pathogenic endpoint of NAFLD, in the metabolic sense, hepatic steatosis is a result of an excess accumulation of lipids within the liver mainly from an overconsumption of fat and/or carbohydrate [1,2]. Dietary fat and carbohydrate vary considerably in the composition of the fatty acid species and sugar molecules. In this review, a high-fat diet refers to a diet containing 30–60% calorie as fat based on long-chain (C16–C20) fatty acids (20–30% saturated and 70–80% unsaturated) in animal studies used to resemble an overconsumption of fat or oils in humans [12,13]. Likewise, the described high carbohydrate in animal models refers to a diet/drink containing high fructose (20–30% in calorie) or sucrose (30–50%), which represents a high intake of soft drink or sweetener in humans [13,14]. Table 1 shows the characteristics of NAFLD in different protocols of feeding with high fat, high fructose, a combination of both, or supplemented with cholesterol. Although all of these diets cause hepatic steatosis, they present different characteristics in the progression of NAFLD to NASH. Because dietary fat supplies exogenous fatty acids to the liver, whereas high carbohydrate (fructose) drives the de novo synthesis of fatty acids within the liver [2], autophagy and ER stress respond differently.

Chronic feeding of rodents with a high-fat diet is well reported to induce obesity associated hepatic steatosis as a manifestation the metabolic syndrome in liver [15,16]. For example, rodents fed a high-fat diet (30–60% fat) display significant hepatic steatosis associated with the features of the metabolic syndrome, including obesity, hyperglycaemia and insulin resistance. Chronic feeding with high-fat diet can upregulate the expression of pro-inflammatory genes and result in hepatocyte ballooning. As shown in Table 1, the changes of NAFLD induced by high-fat diet alone (up to 30 weeks) do not progress to a full range phenotype of NASH with histological inflammation and fibrosis as defined in humans [12,17,18]. Interestingly, addition of cholesterol (0.2–2% *w*/*w*) to a high-fat diet significantly progress hepatic steatosis to a characteristic NASH (including histological inflammation and fibrosis) [19,20,21,22] because cholesterol potentiates the activation of macrophages and hepatic stellate cells to induce inflammation and fibrogenesis [21]. Additionally, cholesterol enlarges the lipid droplets and blunts mitochondrial respiration in high-fat diet-induced NAFLD [21,22].

In humans, the high intake of fructose is positively correlated with the prevalence of insulin resistance and diabetes [23,24]. Accumulated evidence indicates that a high intake of fructose in humans [14,25,26,27] and experimental animals [25,27] is closely related with the development of NAFLD including fibrosis. In mice, the additional intake of fructose during chronic high-fat feeding can also produce typical NASH phenotype beyond hepatic steatosis [13]. It has been shown that addition of fructose and sucrose in drinking water (45 g/L, 50%:50%) activates macrophages and generates considerable degree of fibrosis in high-fat-fed mice, presumably by inducing oxidative stress, which do not occur with high-fat diet feeding alone [18]. There are clear implications (as shown in Table 1) for the involvement of multiple mechanisms in the development and progression of NAFLD initiated by an overconsumption of dietary fat or fructose. Among them, autophagy, endoplasmic reticulum (ER) stress and inflammatory responses [28,29,30,31] have received considerable attention.

**Table 1:** Overview of high fat/high carbohydrate consumption-induced characteristics of nonalcoholic fatty liver disease (NAFLD) in animal models.

Diet	Duration	Hepatic Steatosis	Hepatic Injury	Inflammation	Fibrosis	References
High fat (30–60% kcal)	1–30 weeks	Bioassay TG content ↑ Histological steatosis ↑	Serum ALT or AST ↑ Apoptotic markers ↑ Hepatocyte ballooning ↑ or none	Inflammatory markers ↑ No Histological inflammation	No change in fibrotic markers No histological fibrosis	[9,17,18,32,33]
	30–60 weeks	Bioassay TG content ↑ Histological steatosis ↑	Serum ALT or AST ↑ Apoptotic markers ↑ Hepatocyte ballooning ↑	Inflammatory markers ↑ Histological inflammation ↑ or none	Fibrotic markers ↑ Histological fibrosis ↑	[15,16,32]
High fructose (20–60% fructose)	Up to 25 weeks	Bioassay TG content ↑ Histological steatosis ↑	Serum ALT or AST ↑ Apoptotic markers ↑ Hepatocyte ballooning ↑	Inflammatory markers ↑ Histological inflammation ↑	No change in fibrotic markers No histological fibrosis	[7,10,13,14,33]
High fat, high fructose	12–25 weeks	Bioassay TG content ↑ Histological steatosis ↑	Serum ALT or AST ↑ Apoptotic markers ↑ Hepatocyte ballooning ↑	Inflammatory markers ↑ Histological inflammation ↑	Fibrotic markers ↑ Histological fibrosis ↑	[13,18,32,33,34,35]
High fat, high cholesterol (0.2–2% cholesterol)	12–42 weeks	Bioassay TG content ↑ Histological steatosis ↑	Serum ALT or AST ↑ Apoptotic markers ↑ Hepatocyte ballooning ↑	Inflammatory markers ↑ Histological inflammation ↑	Fibrotic markers ↑ Histological fibrosis ↑ or none	[19,20,21,22]
High fat, high fructose, high cholesterol	25–30 weeks	Bioassay TG content ↑ Histological steatosis ↑	Serum ALT or AST ↑ Apoptotic markers ↑ Hepatocyte ballooning ↑	Inflammatory markers ↑ Histological inflammation ↑	Fibrotic markers ↑ Histological fibrosis ↑	[13,32,34,36]

The data are based on studies in animal models. Histological changes are the pathological endpoint (more severe) phenotypes. Biomarkers alone (less severe) indicate the progression without the endpoint phenotypes. Hepatic steatosis is assessed by increased triglyceride (TG) content and histological steatosis. Hepatic injury is assessed by increased levels of liver enzymes alanine aminotransferase (ALT) and/or aspartate aminotransferase (AST), apoptotic markers (increased mRNA or protein expression of caspase 3, B-cell lymphoma 2 (Bcl-2), and/or increased plasma level or clearing within ballooned cells of cytokeratin 18 (CK 18) fragment) and recognized histologically in the form of hepatocyte ballooning. Liver inflammation is assessed by inflammatory markers (increased mRNA or protein expression of tumour necrosis factor α (TNFα), interleukin 6 (IL-6) and/or inflammasome) and histologically by inflammatory cell infiltration; liver fibrosis is assessed by fibrotic markers (increased mRNA or protein expression of α-smooth muscle actin (α-SMA), transforming growth factor β (TGF-β) and/or collagen 1) and histologically by the presence of collagen fibres.

## 3. ER Stress and Its Possible Roles in the Pathogenesis of NAFLD

ER stress stimulates the unfolded protein response (UPR), which consists of three downstream signalling pathway branches activated by: (a) the inositol-requiring enzyme 1 (IRE1), (b) the PKP-like endoplasmic reticulum kinase (PERK) or (c) the activating transcription factor 6 (ATF6). Liver is an ER stress-sensitive organ, and it is highly responsive to the regulation of these three UPR signalling pathways [29,31]. In relation to development of NAFLD from dietary high fat and high carbohydrate, both IRE1 and PERK branches (not ATF6) are involved but in distinctive patterns [9,10]. Table 2 summarizes the reported activations of the UPR downstream signalling pathways in the development (such as stimulation of lipogenesis and inhibition of fatty acid oxidation) or progression (such as stimulation of inflammatory response and cell death) of NAFLD.

### 3.1. Involvement of ER Stress in the Development of Hepatic Steatosis

Both the IRE1 and PERK branches are activated by feeding with high-fructose diet [7,8,9,10]. IRE1 is an ER transmembrane protein with serine/threonine kinase and endoribonuclease (RNase) domains, which cleaves a 26 base-pair segment from the mRNA of X-box binding protein (XBP1) to generate a spliced form XBP1s. XBP1s acts as a functional transcription factor to induce the downstream events such as ER-associated degradation proteins. When the IRE1 signalling branch is activated by dietary fructose, XBP1s binds to the promoter region of sterol regulatory element-binding protein 1 (SREBP1c) to increase its expression [37]. SREBP1c is a master transcription factor controlling the expression of a number of lipogenic genes, including acetyl CoA carboxylase (ACC), fatty acid synthase (FAS) and stearoyl-CoA desaturase-1 (SCD1) [37,38,39]. Pharmacological inhibition of IRE1 with the chemical chaperone tauroursodeoxycholic acid (TUDCA) and genetic mutation of XPB1 have been shown to block fructose-induced hepatic lipogenesis and lipid accumulation in mice [10,37]. These findings together suggest that dietary high fructose induces hepatic steatosis through stimulating an IRE1/XBP1-SREBP1c-lipogenesis pathway. This mechanism has also been reported to be responsible for mediating postprandial lipid synthesis in the liver [40]. However, the role of the IRE1 signalling branch in response to dietary high fat is less clear because it is activated well after the hepatic steatosis is established (e.g., after 8 weeks of high-fat feeding in mice) [9].

Compared with the IRE1 branch, the PERK signalling branch is activated at a later stage in response to both high-fructose and high-fat diets [7,9,10]. Activated PERK phosphorylates eukaryotic initiation factor 2α (eIF2α), which has also been suggested to increase lipogenesis [7,31]. Although the molecule mechanism is not clear, it is plausible that the PERK signalling branch may play an important role in the maintenance, rather than the initiation, of hepatic steatosis during the pathogenesis of NAFLD. Despite an activation of PERK and SREBP-1c, dietary high fat actually inhibits the de novo lipogenesis in liver [9]. Thus, it remains to be investigated whether the activation of the PERK signalling branch is required for the upregulation of SREBP-1c and about their roles in the increased flux of exogenous fatty acids to liver from dietary fat.

Regarding ATF6, it is not altered by dietary high fat or high carbohydrate (fructose or glucose) [7,9,10]. However, some studies indicate that activation of ATF6 can stimulate fatty acid β-oxidation through upregulation of PPARα, and its downstream targets, such as carnation palmitoyltransferase 1 (CPT1) and acyl-CoA oxidase-1 (ACOX-1) [41,42]. On the hand, ATF6 has been shown to inhibit lipogenesis via downregulating SREBP2 [43]. More definitive studies (such as by genetic deletion of key molecule in this signalling branch) are required to ascertain its role in the pathogenesis of NAFLD from dietary high fat or high carbohydrate.

### 3.2. Involvement of ER Stress in the Progression to NASH

There is evidence to suggest that both IRE and PERK signalling branches are coupled with inflammation by different mechanisms in the progression to NASH [29,31,44]. First, IRE1 can form a complex with TNF receptor associated factor 2 (TRAF2) to stimulate the nuclear factor κB (NFκB) inflammatory signalling by phosphorylating IκB kinase (IKK) and c-Jun N-terminal kinase (JNK) [45]. Activated IKK and JNK also interrupt insulin signalling by phosphorylating the serine residues at the insulin receptor substrate (IRS) [10,29]. PERK can suppress IκBα (an inhibitory protein to NF-κB) to promote the expression of inflammatory cytokines, such as interleukin 1 (IL-1) and tumour necrosis factor-α (TNFα) [46]. Second, SREBP1c has been reported to promote the inflammatory response [29], and as discussed above, this transcription factor is unregulated by both IRE and PERK signalling branches. Third, sustained activation of the PERK signalling branch can induce cell death via C/EBPα-homologous protein apoptosis protein (CHOP) or by downregulating the antiapoptotic protein B cell lymphoma 2 (Bcl2) [47]. It has also been reported that the PERK signalling branch can activate transcription factor 4 (ATF4) to damage cells [48].

**Table 2:** Overview of unfolded protein response (UPR) and autophagy pathways in the pathogenesis of NAFLD.

Signalling Pathways		Molecular Mechanisms	Biological Effects	References
UPR pathways (activation)	IRE1/XBP1	↑ SREBP1c, ↑ ACC, ↑ FAS, ↑ SCD1	↑ lipogenesis	[31,37,38,39]
		↑ IKK, ↑ NF-κB, ↑ JNK ↑ NLRP3	↑ inflammation	[29,45] [46]
		↑ CHOP	↑ apoptosis	[46]
	PERK/eIF2α	↑ SREBP1c, ↑ ACC, ↑ FAS, ↑ SCD1	↑ lipogenesis	[7,9,31]
		↑ IKK ↓ IκB, ↑ NFκB	↑ inflammation	[29] [46]
		↑ CHOP, ↓ Bcl2	↑ apoptosis	[47]
		↑ ATF4, ↑ CHOP	↓ autophagy	[48]
	ATF6	↑ PPARα, ↑ CPT1, ↑ ACOX-1	↑ fatty acid β-oxidation	[41,42]
		↓ SREBP2	↓ lipogenesis	[43]
		↑ SREBP1c, ↑ ACC, ↑ FAS, ↑ SCD1	↑ fatty acid synthesis	[7,49]
Autophagy (inhibition)		↓ Atg7	↑ ER stress ↓ fibrosis	[7,50] [30]
		↑ TNFα, ↑ IL-6 ↑ TLR4 ↑ NLRP3, ↑ IL-1β ↑ NF-κB	↑ inflammation	[51,52] [52] [52,53,54] [52]

## 4. Autophagy and Its Possible Role in the Pathogenesis of NAFLD

**Figure 1:**
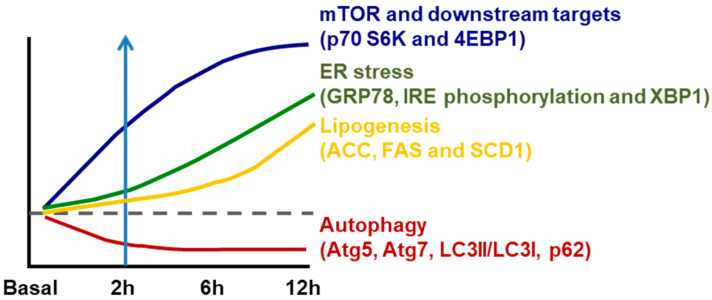
Temporal changes in ER stress associated lipogenesis following mammalian target of rapamycin (mTOR)-coupled autophagy inhibition induced by dietary fructose. However, the activation of mTOR and its downstream p70 S6K and 4EBP1 is parallel with inhibition of autophagy (Atg5, Atg7, LC3II/LC3I and p62) within 2 h of high-fructose feeding. This is followed by increased ER stress (inositol-requiring enzyme 1/X-box binding protein, IRE1/XBP1) (starting from 6 h) and subsequent increase in lipogenesis (acetyl CoA carboxylase (ACC), fatty acid synthase (FAS) and stearoyl-CoA desaturase-1 (SCD1)) (after 6 h). Diagram redrawn based on [7].

ER stress is well recognized to be stimulated by accumulated unfolded/aggregated proteins or inhibited protein glycosylation or disrupted calcium homeostasis within the ER [29]. Recently, it has been shown that ER stress is induced in liver by the suppressed autophagy during high-fructose feeding as shown in Figure 1. Although dietary high fructose activates the IRE1 to promote lipogenesis by upregulating lipogenic proteins, including SREBP-1c, ACC, FAS and SCD-1, restoration of autophagy independently or through mammalian target of rapamycin (mTOR) blocks high-fructose-induced ER stress and lipogenesis [7]. This is very interesting because autophagy (inhibition) has also been implicated in the pathogenesis of NAFLD as shown in Table 2.

Autophagy is well known as a cellular catabolism of breaking down malfunctioning proteins or cellular components including lipid droplets and glycogen [55]. It starts from the formation of autophagosome, which is a cellular fragment targeted for destruction. The autophagosome then fuses with a lysosome to become autolysosome where the targeted proteins and cellular components are degraded via hydrolases from the lysosome. The degraded products (output) of autophagy are released to the cytosol as substrates (such as amino acids and fatty acids), which can be recycled to produce glucose (via gluconeogenesis) and ketone bodies (acetoacetate and β-hydroxybutyrate) via ketogenesis [56,57]. At the early stage of starvation, autophagy of cytosolic proteins constitutes an important source of amino acids. When nutrient shortage persists, autophagy shifts from protein and organelles toward glycogen and lipid droplets as preferred sources to produce glucose and fatty acids to sustain the fuel supply [58,59].

The process of autophagy is regulated at various steps. First, the formation of phagophore (a newly formed double-membrane cup-shaped structure) is controlled by the autophagy-related gene 1 (Atg1)/UNC-51-like kinase 1 (ULK1) complex, which is composed of FIP200, ULK1, Atg101 and Atg13 [60]. Second, during the elongation from the phagophore to form autophagosome, two ubiquitin (Ub)-like conjugation system are required, Atg5–Atg12 and light chain 3 (LC3). The modified cytoplasmic soluble LC3I is transformed into a membrane bounded marker of autophagosome LC3II. Then, LC3II acts as a cargo receptor to recruit adaptor protein p62 to promote the degradation of the engulfed cellular constitutes in the autolysosome [61]. Therefore, ULK1, Atg, the ratio of LC3II/LC3I and p62 are commonly used as markers for the activity of autophagy [7,58].

As autophagy hydrolases lipid droplets (lipophagy), the decreased activity of autophagy can lead to the accumulation of lipids in hepatocytes. Indeed, it has been reported that the lipid-containing autophagosomes is reduced with enlarged lipid droplets in the mice fed a high-fat diet chronically [49]. Inflammation is the main cause of liver damage and can lead to chronic fibrosis of NASH [1,52]. The activation of various pro-inflammatory cytokines (TNFα and IL-6), receptor (toll-like receptor 4, TLR4) and inflammasome (NLRP3) mediates inflammatory reaction and hepatocyte damage, which contributes to the pathogenesis of NASH [51,52,62] (Table 2). The activation of TLR4 is associated with NFκB [52,62] and fatty acids, especially saturated fatty acids, are the activators of TLR4 in macrophages. Macrophages, including liver Kupffer cells, play a key role in triggering liver damage and prolonging the inflammation phase of NASH by releasing mediators of inflammation and fibrosis [63,64]. Autophagy has been suggested to inhibit inflammation by a number of mechanisms (below). It has been reported that under nutrient overload, activated mTORC1 directly inhibits phosphorylation of ULK1 and Atg13 to block autophagy [60], resulting in the development of NASH [11,28].

**Figure 2:**
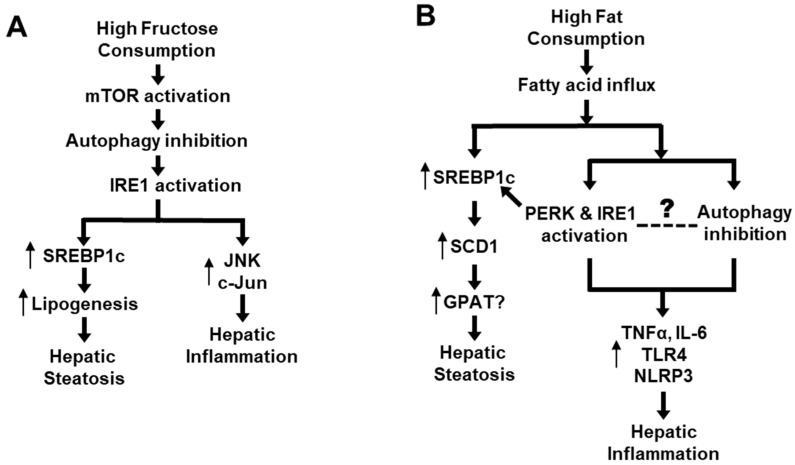
Proposed role of an autophagy-ER stress axis in the pathogenesis of NAFLD. (**A**) Mechanism of high intake of fructose-induced NAFLD via the autophagy-ER stress based on [7,29,31,37,38,39,45]. (**B**) Mechanism of high-fat diet-induced NAFLD via the autophagy-ER stress based on [9,29,31,39,44,46,51,52,53,54].

As previously discussed, high-fructose feeding can rapidly inhibit autophagy in the liver (Atg5, Atg7, LC3II/LC3I, p62) along with activation of mTOR prior to activating IRE1 and upregulating lipogenic proteins (including SREBP-1c) [7]. Further, inhibition of mTOR or restoration of autophagy can block the high-fructose-induced ER stress [7]. As discussed before, activated ER stress not only promotes de novo lipogenesis via SREBP-1c but also interacts with JNK and c-Jun causing inflammation [7,29,31,37,38,39,45] (Figure 2A). Although dietary high fat causes hepatic steatosis mainly by increasing the flux of fatty acids to liver, it also stimulates ER stress and SREBP-1c [9,31,39], which could induce inflammatory response [29,46]. Interestingly, dietary high fat has also been shown to inhibit autophagy activity [44], which may lead to inflammation by various mechanisms such as increases in proinflammatory cytokines (TNFα and IL-6) or activation of receptor TLR4 and inflammasome (NLRP3) [51,52,53,54]. However, we did not find any study on the time-course response between ER stress and autophagy or on their causal relationship during the process of high-fat feeding. Therefore, the causal relationship between inhibited autophagy and activated ER stress in response to dietary high fat remains to be established (as illustrated in Figure 2B). Although addition of cholesterol into dietary high fat exacerbates hepatic steatosis to NASH (as indicated by histological inflammation and fibrosis in liver), there is no evidence of an independent effect of dietary cholesterol on autophagy in this process [19,20,21,22]. Taken together, recent studies appear to suggest an autophagy-ER stress pathway that mediates the development and progression of NALFD due to an overconsumption of dietary high fructose and high fat.

It has been shown that inflammatory cytokines secreted by macrophages play an important role in fibrogenesis [65]. Once NAFLD is diagnosed, whether there is NASH or advanced fibrosis is another important issue to be addressed [66]. Liver autophagy disorder is also closely related to fibrosis [29]. Autophagy plays a role in hepatocyte homeostasis, whereas in hepatic stellate cells, autophagy can stimulate their activation through lipophagy (lipid droplet degradation) resulting in fibrosis [67]. In a mouse model of hepatic fibrosis induced by CCl4 or thioacetamide, knocking out the key autophagy protein Atg7 can alleviate the level of liver fibrosis [29].

Autophagy is closely interactive with ER stress [7,48,50]. ER stress has been reported to regulate the transcription of key autophagy proteins (Atg3, Atg5, Atg7 and p62) through ATF4 and CHOP [48]. This has been well demonstrated in high-fat-induced obese mice where the autophagy in liver is reported to be severely inhibited [50]. This same study shows that when defective autophagy is restored by overexpressing Atg7, the elevated ER stress (the PERK-eIF2α-CHOP axis) is relieved. Of particular note, mTOR is a major upstream regulator of autophagy activity, and mTOR is regarded as sensor to the nutrient status of cells [7,59,60]. Therefore, under the regulations of mTOR autophagy, there is a much more sensitive pathway than previously thought, and it plays a dynamic role in the adaptive metabolism in liver during nutrient fluctuations. Overall, the defective autophagy is involved in the pathogenesis of NAFLD [6] at multiple steps, including ER stress [50], hepatic steatosis [11], the progression to NASH [52] by producing inflammatory factors [29] and activation of the fibrogenic pathway [30].

## 5. Concluding Remarks

**Figure 3:**
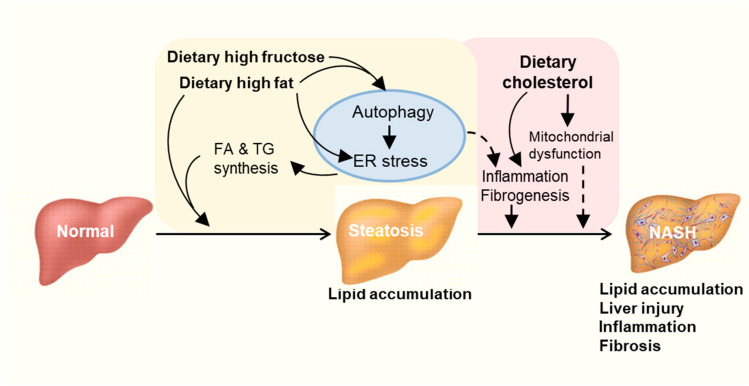
Proposed autophagy-ER stress-inflammation in the pathogenesis of NAFLD induced by overconsumption of dietary fat and fructose. High fat or high fructose consumption can cause excessive lipid accumulation in the liver known as hepatic steatosis. Cholesterol consumption can aggravate hepatic inflammation and fibrosis possibly independent of autophagy and ER stress. Multiple mechanisms are involved in the development and exacerbation of NAFLD including autophagy, endoplasmic reticulum (ER) stress and inflammatory responses. FA: fatty acid. TG: triglyceride.

NAFLD has become a global health problem. Unhealthy dietary habits especially Western diet rich in high fat and carbohydrate are closely associated with NAFLD (Table 1). It is clear now that mechanisms beyond a simple accumulation of lipids in the liver play a key role in the progression of the NAFLD [27]. This review examined the recent literature reports on the involvement of autophagy and ER in the development of hepatic steatosis and its progression to NASH in context of high ingestion of fat and high carbohydrate (fructose). Based on this review, we propose a mechanism of an intrinsically linked axis of impaired autophagy and unresolved ER stress that mediates the pathogenesis and exacerbation of NAFLD from the overconsumption of high fat or carbohydrate as illustrated in Figure 3. Further studies are required to investigate the mechanisms of newly identified drugs targeting ER stress or autophagy pathways in the treatment of NASH. These may reveal novel target for the significant treatment of NASH and related metabolic diseases.
